# LifeLab: Co-Design of an Interactive Health Literacy Intervention for Socioeconomically Disadvantaged Adolescents’

**DOI:** 10.3390/children9081230

**Published:** 2022-08-15

**Authors:** Craig Smith, Hannah R. Goss, Johann Issartel, Sarah Meegan, Sarahjane Belton

**Affiliations:** School of Health and Human Performance, Dublin City University, D09 NA55 Dublin, Ireland

**Keywords:** adolescence, health literacy, process evaluation, formative evaluation, health education, co-design, qualitative

## Abstract

Low socioeconomic populations, when compared to more affluent groups, are at greater risk of initiating risky behaviours and consequently developing health complications. Health literacy has been identified as a possible means to improve and sustain positive health behaviours, with adolescence being a time point when such behaviours can be embedded. To develop a meaningful health intervention, it has been recommended that relevant stakeholders be included in the design phase. This formative evaluation study was the second phase of co-design of an engaging health literacy intervention ‘LifeLab’ with, and for, socioeconomically disadvantaged adolescents in Ireland. In Spring 2021, a series of co-design workshops (*n* = 17) were facilitated with a convenience sample of adolescents from socially disadvantaged areas (*n* = 22) to gather their perceptions, feedback, and suggested changes on the LifeLab learning activities that had emerged from Phase 1 of the work. The data was analysed using reflexive thematic analysis, resulting in the development of three themes: (i) preferred learning engagement strategies, (ii) practical and logistical considerations and (iii) ideas for LifeLab content. The results highlight the value in adopting a participatory approach, as participants offered an array of suggestions and details to maximise the potential for LifeLab to be contextually relevant and engaging; suggestions which will directly inform the development and implementation of the intervention.

## 1. Introduction

Lifestyle behaviours, such as dietary habits, insufficient sleep, substance misuse and low levels of physical activity are becoming increasingly prevalent, resulting in a continuous rise in non-communicable diseases (NCDs) [1,2,3]. This issue is more pronounced in low socioeconomic populations when compared to more affluent groups, with research demonstrating that socioeconomically disadvantaged populations are more likely to engage in unhealthy behaviours and are consequently more likely to experience lifestyle-related health complications [4,5,6]. To tackle these growing health inequalities, targeted health promotion and health education initiatives have been recommended [7].

Adolescence has been identified as a period when health promotion could have a lasting impact, given that the health knowledge, values and behaviours that are developed during this life-stage are often embedded and track into adulthood [8]. Unfortunately however, it can often be risky behaviours that are initiated and established during adolescence [9]. In Ireland, young people from socioeconomically disadvantaged populations are at a greater risk of initiating poor lifestyle behaviours, leading to disparities in health-related outcomes such as obesity and overweight [10]; substance misuse [11]; and health-related fitness [12]. Health literacy (HL) has been identified as a key determinant of health, and a possible means to improve and sustain healthy lifestyle behaviours in young people [13]. HL is defined by the World Health Organisation (WHO) as the cognitive and social skills which determine the motivation and ability of an individual to gain access to, understand and use information in ways which promote and maintain good health [14]. For health promotion practitioners, HL is conceptually attractive as it is a personal asset that can be developed through educational interventions [15]. The concept of HL has been identified as an empowering resource that can guide an individual to make informed health-related decisions [13], with studies reporting a clear association between those with high levels of HL, healthy behaviours and positive health outcomes in children and adolescents [16]. In contrast, it has been reported that financial deprivation and low social status are the strongest predictors of low HL [17], a possible contributor to high uptake in risky behaviours in these populations. Despite these findings, and the fact that HL is a modifiable factor that can be influenced by education [16,18], there is a lack of interventions explicitly targeting HL in adolescents, particularly from socioeconomically disadvantaged populations [19]. The school setting has been suggested as an ideal environment for health interventions given the potential reach and the volume of time that most young people spend in schools [20]. Furthermore, research suggests that health promotion interventions in youth from socioeconomically disadvantaged populations are most successful when they include an educational component [21], as education has been shown to improve attitudes, develop HL and improve health behaviours in adolescents [22].

Despite the clear rationale and justification for school-based health promotion, the effectiveness of such interventions in improving health behaviours has been equivocal [23]. It has been observed that many studies focus solely on the effectiveness of an intervention, often ignoring the reasons behind why it was successful or not [24,25]. By including a thorough evaluation of an intervention, such as a formative evaluation, the influences on the effectiveness of the intervention can be assessed [26]. Formative evaluation is often deployed during the design phase of a health intervention to optimise the efficacy of the intervention, to assess the strengths and weaknesses, and to ensure that the needs of the targeted population are met [27]. Without formative evaluation, interventions may fail to translate into meaningful health outcomes, with the reasons for that failure remaining unclear, thus providing little guidance for future interventions [28].

Adopting a participatory approach allows for research to be carried out ‘with’ rather than ‘on’ participants, increasing the likelihood of developing an intervention that is contextually relevant, feasible and sustainable [29]. Evidence also suggests that school-based interventions should use integrative and comprehensive approaches to health promotion that target the participants’ behaviours, attitudes, and community [30]. Yet, many interventions fail to involve the relevant participants in the intervention design, which can result in a lack of alignment with the core principles or health issues of the target population [31]. Ensuring interventions are tailored to the needs of the targeted individuals is particularly important when it comes to low socioeconomic populations, as health interventions targeting, but not specifically designed for, these cohorts often have poor uptake and make little impact, which can lead to a widening of health inequalities [32]. This has resulted in a recent push towards including relevant, non-academic partners in the co-design of interventions, partners who have typically been excluded from this phase but can significantly contribute to, and benefit from, health-related research, and in turn reduce research wastage, increase overall impact and address inequities [32,33,34].

This project followed the Ophelia (Optimising Health Literacy and Access) framework [35], which aims to involve a range of local stakeholders in developing HL interventions that are based on the diverse HL strengths and weaknesses of the community/cohort in question. The Ophelia process includes three phases (identifying local strengths, needs and issues; co-design of interventions; implementation, evaluation and ongoing improvement) and is underpinned by eight key principles, including being equity driven, outcomes focused, and adopting a co-design approach [35]. In line with the Ophelia framework [35], previous work on this project has identified local HL strengths and weaknesses through the development of a series of vignettes generated from locally collected data, providing real-life examples of the diversity of the targeted adolescent population [36]. Using these vignettes, initial co-design workshops with local socioeconomically disadvantaged post-primary students and school staff identified key health topics (food choices, mental health and wellbeing, physical activity and sedentary behaviour, sleep, and substance misuse) and potential health-related capacity building actions that could be implemented [37]. These actions included: interactive, applied and relevant activities; fun and engaging off-site delivery; and education around the short and long term impact of health behaviours [37]. Using the information gathered thus far from the aforementioned previous research on the project [35,36] and data from the literature [19], the structure of the LifeLab intervention, along with draft ideas for the learning activities in LifeLab, were preliminarily designed by the project team (to allow for further input from young people); which provided the starting point for the current phase of research.

Building on this previous work, this study aimed to investigate adolescent’s perceptions of the specific LifeLab learning activities which emerged from Phase 1 [37] in order to guide refinements and inform the final intervention structure prior to a pilot trial and thereby improve the potential efficacy of this intervention.

## 2. Materials and Methods

It is of note that this formative evaluation study was carried in the midst of the COVID-19 pandemic, a time when HL was perhaps more important than ever [38]. HL capacities allow individuals to be well-informed about public health recommendations in relation to COVID-19, and to act in ways to best reduce the associated risks of contraction and transmission of the virus [39]. Critically, there is a major concern for health equity, with the greatest effects of the pandemic likely to fall on disadvantaged populations [38]. The COVID-19 pandemic resulted in closures of schools for large periods of time in Ireland, requiring much of this study to be carried out using methods that complied with government, university and school guidelines and regulations. This research was conducted at a time when Ireland was in a strict lockdown (January–May 2021). These restrictions included the closure of non-essential retail, restaurants/dining, travel restrictions and prohibition of social gathering outside of an individual’s “social bubble”. Schools had pivoted to online teaching and only reopened for in-person lessons from March 2021 onwards.

### 2.1. Participants and Recruitment

Ethical approval for this study was granted by the institutional research ethics committee [DCUREC/2020/048]. Schools, which had been previously involved in the project were asked if they would like to participate in this phase (Phase 2) of the project. There were no strict inclusion or exclusion criteria, but all schools were part of the Irish Department of Education’s ‘Delivering Equality of Opportunities in Schools’ (DEIS) action plan [40]. One mixed-gender school (‘School A’ from here on), who previously participated in Phase 1 of the research [37], continued their involvement in Phase 2 of the project. While three other Phase 1 DEIS schools were also invited to participate, they declined due to time and teacher availability; directly citing issues related to the COVID-19 pandemic. To ensure a breadth of involvement in the co-design process, three additional mixed DEIS schools were recruited. School contacts were asked to identify students with a range of diverse backgrounds and interests, across the three years of Junior Cycle education (1st to 3rd year; age 12–16 years), who would be willing to join the workshops. Nine participants from one 1st year class in School A (age 12–13, who had not previously been involved in Phase 1) agreed to take part, and a convenience sample of participants (*n* = 13) aged 12–15 from the three additional mixed-gender DEIS schools were recruited to form a *Youth Health Forum* (YHF). This brought the total number of adolescent participants involved to 22. A flowchart detailing the recruitment process has been included in the Supplementary Materials (Figure S1). The project was explained to all students by the school project contact, and they (and their parents/guardians) were given a plain-language statement and consent/assent forms to complete.

### 2.2. Draft LifeLab Activities

Using data gathered from previous co-design workshops with this cohort [37] and discussions with experts in the field, the LifeLab learning activities, or ‘stations’, were roughly planned and mapped out by the project team. This initial LifeLab intervention map proposed to include two separate visits for school class groups to the physical ‘LifeLab’ onsite at Dublin City University (DCU, ‘LifeLab 1’ and ‘LifeLab 2’), in conjunction with school-based learning activities to be implemented by teachers under the ‘Wellbeing’ curriculum, before, in-between and after each visit. Following the findings of Goss et al. [37], LifeLab 1 and 2 had the goal of developing HL through a hands-on interactive experience, where post-primary students from participating schools would visit the DCU LifeLab to explore and learn about common health issues previously identified by adolescents and school staff [37]. Specifically, LifeLab proposed to provide a carousel of learning activities that the participants could explore, with each activity focused on a specific health area that had been identified by the teachers and students in Goss et al. [37]; food choices, mental health and wellbeing, physical activity and sedentary behaviour, sleep habits, social and environmental factors, and substance misuse. Although the overall structure of the intervention, and draft ideas for the learning activities had been preliminarily designed based on the Phase 1 research, a formative evaluation of the learning activities was required, along with a further co-design opportunity with the key adolescent stakeholders, to finalise the design and implementation of the LifeLab intervention for a pilot trial.

### 2.3. Procedures

A total of 17 co-design workshops (nine with the YHF and eight with School A) were conducted separately between January–May 2021, to discuss the LifeLab activities individually. As a result of the COVID-19 pandemic and the consequential closures of schools in Ireland, most (13 workshops) of the workshops were held over Zoom or Microsoft Teams (depending on the school’s preference), with latter workshops being held in a classroom in the school when COVID-19 restrictions were eased (four workshops). Online focus groups have become popular in recent times, with many stating that the quality and quantity of data obtained is comparable to data captured in face-to-face focus groups [41]. To ensure that the online focus group dynamic was as similar as possible to that of traditional-style focus groups, extra emphasis was placed on establishing a creative, synergistic and non-inhibiting environment by encouraging group discussions and regularly asking additional questions to clarify participants’ views [41].

The facilitators (CS and HG), who were experienced in HL and health education in post-primary students, followed a structured protocol. Typically, the co-design workshops lasted roughly 40–60 min, where one activity was displayed on a PowerPoint presentation. The facilitators described the idea for the learning activity to the participants, and in most cases (where possible), the participants completed an adapted online version of the activity to get a sense of how it would work. Upon completion of this and after confirming that everyone understood the activity fully, the participants described and evaluated the station idea using an anonymous polling platform (Vevox, 2021 Auga Technologies Ltd., Godalming, UK). Firstly, participants were asked to use their own words to describe the idea for the activity, with their suggestions displayed as a word cloud only after everyone had contributed as many/few words as desired. This was followed by questions using a 5-point Likert scale to evaluate how interesting, fun, engaging and exciting the participants found the idea for the activity. The primary purpose of this initial element of the workshop was to get the participants thinking about the activity and their perceptions, before delving into a deeper analytical discussion. This process followed the method discussed by Morrison-Beedy et al. [42], which suggests introducing the level of questioning gradually to develop rapport within the group and to allow for deeper discussions around a given topic. Building on the participants’ responses, the activity was then discussed in more detail using a semi-structured question guide. As well as expanding on the responses from the polling platform (e.g., Why did you find the activity so interesting/fun/engaging/exciting?), the question guide aimed to ensure that the practicalities, acceptability and equity of each activity idea was thoroughly discussed. The final, and perhaps most critical, element of the workshops involved inviting participants to share and discuss their ideas on how the station could be refined, improved, or enhanced, or to offer completely new ideas for learning activities, if they had any. All co-design workshops were audio-recorded using a digital dictaphone, and reflective diaries were used to record insights and ideas by the two facilitators and a critical observer, which were discussed immediately after the workshop.

### 2.4. Analysis

Reflexive thematic analysis (RTA) [43] was conducted to analyse the data gathered. RTA is about the researchers’ reflective and thoughtful engagement with their data and their reflective and thoughtful engagement with the analytic process [44]. The findings, therefore, primarily reflect the first author’s (CS) analysis of the data. Aligning with the principles of RTA, the co-design workshops were conducted with flexibility and fluidity to reflect a real-life conversation, providing the researcher with the freedom to be responsive to the participants’ feedback and input. This approach aimed to create a relaxed environment and allowed for an in-depth analysis of the participants’ perceptions of the LifeLab learning activities and intervention more generally. An inductive coding approach was adopted, focusing on deriving semantic and latent codes and generating themes linked to the data acquired. An experiential orientation to data interpretation was adopted to explore the participants’ contextually situated experiences and perspectives on the intervention and was underpinned by a constructionist epistemology. As such, the interpretation of meaningfulness was highly influential in developing codes and themes [43].

The data was analysed using Braun and Clarke’s [41,42] six-phase approach to RTA. First, the data was manually transcribed verbatim to Microsoft Word. Each transcript was then read multiple times to ensure familiarity and understanding of the data before systematic coding began. After the full dataset was coded using a qualitative analysis software (QRS NVIVO-12), the initial themes were generated from the codes. The initial themes were then reviewed, developed and structured. Throughout this process, the themes were refined, defined and renamed until they were deemed appropriate. The reporting phase was completed after the codes and themes developed.

Throughout the data collection and analysis, the first author (CS) kept a reflexive journal to record key information, interpretations and insights that were constantly reflected on during the analytical process. Due to the reflexive and interpretive nature of RTA, the first author (CS) predominantly analysed the data, with co-authors (HG and SM) assisting by sense-checking analysis and offering suggestions and alternative interpretations. Resultantly, the findings predominantly reflect the first author’s analysis of the data and were challenged by the co-authors, leading to rich, thorough and reflexive analysis [43].

## 3. Results

Using the polling platform, the most common words used by the participants to describe the LifeLab activities included: exciting, interesting, fun, cool, innovative, and different. The quantitative scores from the Likert scale evaluation were not formally analysed. Responses to the Likert scale evaluation questions, however, were used to facilitate detailed discussions around the LifeLab activities, specifically the factors that they most and least enjoyed.

Following the RTA of the data gathered, three themes, with subsequent sub-themes, were generated: (i) preferred learning engagement strategies, (ii) practical and logistical considerations and (iii) ideas for LifeLab content. Table 1 presents all themes and sub-themes with exemplar quotes.

### 3.1. Theme: Preferred Learning Engagement Strategies

During the co-design workshops, the participants highlighted specific strategies that they felt would enhance the engagement of young people with the LifeLab activities. These strategies were recommended as methods that could be adopted to create appealing, exciting and enjoyable health-related learning activities. The suggestions included incorporating healthy competition; interactive tasks; problem solving to various stations; and ensuring that there was variety and choice across and within learning activities.

#### 3.1.1. Healthy Competition

The participants identified healthy competition as a key strategy to engage and interest young people in learning about health. This was discussed across various activities with one YHF participant suggesting that “everyone would want to take part more if there was, like, competition involved” and that it would make it “more fun for everyone”. Despite advocating for an element of competition in the learning activities, participants were also wary of its potential pitfalls, stating: “It could be fun. But at the same time, I wouldn’t want [to be] the people that don’t win. Like, I wouldn’t want them to kind of disengage from that” (YHF participant).

This highlighted the participants’ awareness of ensuring that the element of competition was fair and that it didn’t result in some individuals being excluded from the activity and learning. This was emphasised by one participant who stated that “it’s important that if we were gonna do some competition that it’s still fun and relaxing and enjoyable”, and that the form of competition is crucial to consider:

“I think it might be better to have a point system so that if you don’t get an answer, you don’t just stand, like, on the side-lines or in like a corner waiting for everyone else to be finished. And like, if the point system was fair, it would be more fun because then you wouldn’t have to be worried about, like, getting knocked out” (YHF participant).

#### 3.1.2. Interactive Tasks

The participants identified interactive tasks as an engaging (and informative) strategy in LifeLab, particularly when they were fast paced. This was clearly articulated when the YHF participants commented the following after completing an idea for an activity: “it was fast and interactive, you didn’t have time to get bored” and “I liked how they were short and sweet, but they still have a little bit of information, so you learn something from it”. Furthermore, bringing the learning to life through use of a familiar or established game was well received by the participants, with one participant stating that they enjoyed the idea of creating “a game that everyone knows into something that, like, links in with health and wellbeing” (YHF participant). This was particularly the case when the participants could physically complete a task during the game:

“I think for the puzzle part, as long as it’s not like on paper and it’s interactive or you can physically do it together… It will be more memorable and more interesting and get people more involved” (YHF participant).

These insights suggest that the participants wanted to avoid being stationary during the learning (as may be typical in a school classroom), and that to elicit a “positive reaction” from young people, learning activities which incorporate an element of physical activity or movement would be more effective in maintaining engagement: “If it was just like sitting down, like, on iPads and just playing Kahoot, I think they’ll be boring” (YHF participant).

#### 3.1.3. Problem Solving

Incorporating an element of problem solving into the learning activities was a popular idea among the participants. During a discussion on developing a learning activity to explore food choices, a School A participant suggested making the activity into a game that would involve participants “trying to work it out”. This point was emphasised by another YHF participant, who said: “I feel like if there’s almost like a complicated puzzle, like where a group of people would be able to figure it out. That’s what makes it really fun, I guess”. In the workshops, the participants discussed how they would like to work in “teams” to complete problem-solving activities, rather than “having the spotlight on one person”. Another reason for the participants preferring to work as a group was that they could seek out help from peers: “If one person was confused…they could all kind of help each other out”, which would make the activities “more fun” and “less stressful” (YHF participants).

In addition, the participants from School A felt the level of difficulty was an important factor to consider. When discussing ideas for an activity on sleeping habits, the participants mentioned that the questions originally designed for the activity were “very easy” and they asked for “harder questions” to avoid the answers being “a little bit predictable”, causing the activity to go by “faster because it is very easy”. In contrast, participants were keen to avoid the activities being “too difficult to think about” as a result of asking too many questions on a given topic and they acknowledged it would be “difficult to kind of hit the balance” when it came to creating educational content for the activities.

A common observation from the participants, across multiple learning activities, was the importance of including an element of “time pressure” to the problem solving to maintain engagement, with some claiming that the “time crunch kind of makes it” and that: “If the timer wasn’t there I think everyone else would kind of be bored of waiting for them to answer” (School A).

Participants even suggested using the time pressure to create competition between participants: “It’d be like really interesting to see who got the fastest time and who got the slowest time” (YHF participant).

#### 3.1.4. Providing Variety and Choice

The workshop participants emphasised the importance of providing variety to engage young people, with multiple participants commenting on how they enjoyed the fact that all activities were different:

“It stands out, but in a good way because you kind of need variety… if you have too many similar games, people might get bored of them after a while… so I think it’s really good” (YHF participant).

Furthermore, offering choice to participants was deemed crucial as “it is important to have something to engage all types of people”. This was particularly evident during an activity focused on highlighting the importance of sleep hygiene, which involved some students listening to meditative audio while sitting in a ‘relaxation zone’. One student commented on this idea by saying:

“A lot of people do meditation because it helps them, but they do it by choice. Meanwhile, if someone just turns on a video and says sit still now and listen and do this. Like, for some people doing nothing is just really hard and it makes them more stressed then relaxed, so it has the opposite effect” (YHF participant).

This feedback emphasised participants’ recognition that not all health-promoting techniques are effective for everyone, along with the importance of providing a suite of options for participants to choose from when demonstrating, and educating on, healthy techniques: “You’d probably have to ask someone…who is in the station (learning activity) which one would they prefer” (School A participant).

### 3.2. Theme: Practical and Logistical Considerations

In order to ensure that the LifeLab intervention was as enjoyable as possible, numerous practical and logistical considerations were highlighted by the participants during the co-design workshops. These included considering the amount of time spent on each activity; the number of students completing each activity at a given time; and creating an appropriate physical space for the participants. These considerations are strongly linked with, and are indeed complementary to, the learning engagement strategies detailed above.

#### 3.2.1. The Amount of Time per Learning Activity

During the co-design workshops, participants discussed the amount of time that they would ideally spend on each activity. In conjunction with this, the students highlighted their desire for fast-paced activities; they also emphasised the importance of keeping the activities short enough to maintain engagement and interest:

“I don’t want to have to play it until I get bored. I think it would be good to cut it off at an exciting point so that we look back on it happy rather than like ‘oh it went on and on’” (YHF participant).

When asked specifically about the amount of time they would want to spend on each activity, the responses varied greatly between participants, and also depended on the activity in question being discussed. One student suggested completing an activity for “two hours” while another suggested “six minutes”. On average however, participants recommended staying on one learning activity for roughly 10–30 min before moving to another, with key feedback highlighting the importance of not “staying on one thing for too long” and keeping the activities short enough to avoid disengagement or boredom.

#### 3.2.2. The Number of Students Completing a Learning Activity at One Time

During a discussion around the number of students that would ideally complete a single learning activity at one time, the participants were keen to ensure that the group size was not too large: “I feel like if it is too big some people might not get the experience of it. I’m thinking like definitely less than ten [people]” (YHF participant). In order to guarantee that everyone has the chance to benefit from the activity, others suggested the number per group should be even less: “I think up to five people is good because it gives everyone a chance to speak if they want to” (YHF participant).

Adding to the concern for LifeLab attendees feeling excluding or “left out” of the learning, the participants who recommended working in teams during activities strongly suggested that such teams comprise of “pairs” or “two to three people”, as “it’s easy to get a smaller group to work together on one thing” and would still allow for participants to “ask their friends for help”, if needed.

#### 3.2.3. Creating an Appropriate Physical Space

The importance of the physical space in LifeLab was highlighted during the co-design workshops. This was particularly evident when it came to activities where the participants had the opportunity to practise mindfulness or relaxation techniques. For such activities, participants wanted to ensure that the physical space created the appropriate environment to engage in the intended activity: “So for example, the meditation one, like little lava lamps, or you know them lights that make them colours, stuff like that would be really cool” (YHF participant). Another participant suggested including “bean bags and blankets” for the LifeLab attendees to create a “relaxing type of place”. They even commented on the impact of the colour schemes adopted, stating that in order for LifeLab to create an “aesthetic” and “visual” appeal, the activities and LifeLab space should include “warm tones or even bright colours… like green… to make it like refreshing or calming” (YHF participant).

### 3.3. Theme: Ideas for LifeLab Content

Throughout the discussions on the specific LifeLab learning activities, the participants provided feedback on not only the methods to engage young people in health-related activities, but also on the content itself. The feedback highlighted the importance of including learnings that were meaningful and helpful to young people in their context.

#### 3.3.1. Relating the Content and Learnings to Real Life

Participants were keen to have content and learnings in LifeLab that they felt were relatable and could be applied to their own lives. As part of many of the LifeLab activities discussed in the co-design workshops, contextually relevant vignettes (developed in Phase 1, [36]) were employed to allow participants to freely discuss health topics and behaviours, explore their own health concerns, and develop solutions to real-life health challenges. The participants engaged with the characters very well and provided positive feedback on their use:

“I liked the long one—what was it called?—where you read a little bit [about the vignette] and then you have to give them advice. I like that one because it’s something that you can almost make up on the spot, but it just makes you think a little bit more” (School A participant).

When asked about how we could improve specific activities, the participants replied with “add more scenarios [vignettes]” and add “different people [vignettes]”. The purpose of the vignettes, which was to engage the young people and allow them to relate the learnings back to their own life in a non-judgmental manner, appeared to be effective. This was demonstrated when one participant described how the process of discussing and analysing the vignette’s poor sleeping habits, might cause a young person to re-evaluate their own sleeping habits: “So that would maybe, like, make them be like, you know, oh, maybe I shouldn’t be doing that” (School A participant).

The participants also suggested directly analysing their own health through LifeLab activities. One activity discussed in the co-design workshops, which was aimed at developing HL around sleeping habits, observed how different behaviours prior to sleep impacted brain and heart-rate activity, and subsequently affected one’s ability to obtain an adequate night’s sleep. Rather than just visualising the impact of these behaviours on a computer screen, and possibly only seeing a peers’ physiological response, one member of the co-design workshops suggested:

“I would probably enjoy it if you could like print out like what information you got from like the brain scan and the heart-rate monitor, I feel like that would be really interesting… so you can see how your brain reacts to what you were doing and how your heart-rate reacts” (YHF participant).

Participants wanted to “learn things” from LifeLab that they could “put into their daily lives”. This was evident when discussing an activity aimed at food choices, where they stated: “I feel like it’s also a good idea because then people could think about what food they should and shouldn’t consume, and like what they can do to change their diet and like how they can fix it”.

#### 3.3.2. The Influence of Social Media on Young People

Social media and its daily influence on young people came up in conversation across many of the workshops. The participants mentioned how many of the interactions between peers in school are based on current trends on “TikTok and Instagram”:

“Ninety-five percent of the jokes that me and my friends make, and people at school that I hear, are from TikTok. It has such a vast spreading, like if a TikTok goes well, everybody knows about it and everybody gets the joke… so, I feel like that influences people so much nowadays” (YHF participant).

The participants went on to describe how it is not just entertainment content that young people are engaging with on social media, often they use these platforms for health-related information:

“Like some doctors and nurses are on TikTok and they’re trying to inform people of, like, some health conditions or like even what to do if you’re feeling kind of sick or something” (YHF participant).

According to the participants, the health information shown on these outlets is mostly focused on eating habits and exercise, where influencers post “workouts” and “what to eat in a day” videos. The participants acknowledged that often these influencers are attempting to “make themselves look perfect” and that the way they look and behave is often not “normal”, yet they still recognised that their content can often be detrimental to young people’s health:

“Social medias, and like basically photoshopped images of the perfect body, can affect a lot of people’s mental health” (School A).

#### 3.3.3. Lifestyle Behaviours and Their Impact on Health

The co-design workshops highlighted the need and desire for education around lifestyle behaviours and the potential impacts that they can have on the body and health more generally. The participants articulated this by asking for more content on “the consequences” of lifestyle choices. In particular, they were interested in learning about “what they’re doing right and what they’re doing wrong” and how these behaviours can alter quality of life:

“It was really exciting getting to see like how your decisions would, I know you haven’t seen it yet, but how your decisions would affect something that you can’t see on the outside…But like they have an influence on your life” (YHF participant).

Participants also identified the body as something that they would like to learn more about, specifically how the body changes as a result of lifestyle choices, or how we can “ruin the body”. This was mentioned after trialing an activity aimed at highlighting the impact of substance misuse, where the students had to match up a body part with the appropriate behaviour (e.g., unhealthy lungs, with cigarette smoking):

“I wouldn’t really know how the body looks overall healthy and how it looks like not healthy, so it’s going to be very interesting to see what I would think is, like, a healthy looking body and what I wouldn’t think…and I would learn about what the body should look like and what the body shouldn’t look like, you know, so it’s very educational at the same time” (School A participant).

Furthermore, not only did the young people want to know how the appearance of the body might alter as a result of lifestyle choices, but they also wanted education around changes to the how the body functions as a result of specific behaviours:

“You could kind of go and show some information about, like, your lungs and like, I don’t know, I guess how they work more efficiently maybe…you know the people who smoke, their lungs wouldn’t be very healthy…and then people who exercise, they would have a better lung capacity” (School A).

## 4. Discussion

The research aim of this study was to formatively evaluate, and co-design future content, of ‘LifeLab’; an out-of-school HL engaging learning experience for socioeconomically disadvantaged adolescents. To achieve this aim, co-design workshops were carried out with a diverse group of adolescents from DEIS schools, which were focused on obtaining feedback from the young people on the LifeLab learning activities and suggested refinements in order to ensure maximal acceptability and efficacy of the intervention going forward. The study findings offer a valuable insight into young people’s perceptions of HL education; specifically, methods which may be used to engage young people, practical considerations for implementing an HL intervention, and the health-related content that young people in this context feel is meaningful and important to learn about.

Regarding the engagement strategies, the participants’ feedback highlighted the appetite for an intervention which is hands-on and interactive. This aligns with literature suggesting that interactive learning outside of the school classroom provides memorable experiences that can track into adulthood and have lasting impacts on health behaviours [19,45,46,47,48]. Moreover, interactive learning activities that incorporate context-specific learning have been shown to improve adolescents’ HL and potentially health behaviours [45,48]. This has been already modelled by LifeLab in Southampton; an innovative, hands-on, science-based approach targeting adolescents’ HL through scientific knowledge targeting human biology, lifestyle behaviours and critical thinking [49]. Findings from LifeLab Southampton’s pilot studies have demonstrated that such an intervention can have a positive impact on adolescents’ health-related attitudes and knowledge [49]. Adopting similar pedagogical approaches in the current context will provide evidence on whether similar findings are generalisable to socioeconomically disadvantaged adolescents in Ireland.

The use of competition in education is a contentious topic, with some stating that by adding a competitive element the focus shifts away from the learning process and onto the competitive goal [50]. Others, however, are strong supporters of its use. For example, Lawrence [51] believes competition encourages active learning and increases motivation, while Fasli and Michalakopoulos [52] state that it acts as an incentive for students to put in more effort and can allow for academically weaker students to engage in an activity. ‘Healthy competition’ has been defined as a short activity where the focus is on the learning process rather than the results, and the outcomes are trivial [53], with team-based competition posing less risk for the task to become solely goal focused in young people [54]. Furthermore, it has been recommended that in order to facilitate healthy competition in education, it must be conducted over a short period of time, provide a range of topics and tasks, allow everyone the opportunity to succeed, allocate a clear value to the learning process and quality, and aim for enjoyment [54]. In line with the above, suggestions from the participants in this study support that including short bouts of healthy competition within learning activities may provide a viable method to capture young people’s attention to maximise engagement and facilitate their learning of HL-related content.

Problem-Based Learning (PBL) is a method of learning that often involves students working collaboratively to define and solve a problem while developing communication and critical-thinking skills [55,56]. Though the term ‘PBL’ was not used by participants, PBL strategies were often central to suggestions made by participants for enhancing student engagement with the LifeLab learning stations. Savery [56] defines it as a learner-centred approach that empowers individuals to interrogate an issue and apply knowledge and skills to develop a viable solution. This method allows participants to acquire knowledge through peer-learning rather than depending on the teachers to disseminate information. Instead, teachers can act as facilitators of learning who can scaffold participants with effective questioning and guidance [57,58]. To allow for productive and constructive peer-learning, the participants requested that the groups (teams) were small enough to allow for all participants to have an opportunity to maintain their desired involvement in the task, a suggestion echoed in the literature [59,60]. The aims of problem-based learning overlap with goals of HL development, where the enhancement of higher-order skills, such as comprehending, reasoning, critical thinking and application, are critical [14]. Moreover, given the self-directed style of problem-based learning, whereby students use reflexive thinking and have the opportunity to develop soft skills required for everyday life, it appears to be a fitting method to educate on real-world health-related situations.

In order to maintain engagement in the LifeLab activities, the level of difficulty needs to be considered, according to the co-design workshop participants. Shields and Funk [54] stress the importance of finding the balance in tasks, in that they need to be both challenging and achievable. They explain that when tasks are deemed too easy by young people, it results in boredom and disengagement, but when tasks are too difficult, young people will become frustrated and lose interest. In order for LifeLab activities to maintain participant engagement, the level of difficulty of the content must be appropriate for its intended target audience. Similarly, the amount of time the participants wanted to spend on each activity was a talking point across the workshops. The participants emphasised that they wanted enough time to be able to complete the tasks fully and enjoy the competitive element but mentioned that if the activities were repetitive or took too long, they would lose interest. For most of the participants attending the co-design workshops, this appeared to be anywhere between 10–30 min, but for some it was significantly less. This highlights the need for flexibility in the design of engaging health interventions for young people and the importance of tools or techniques to re-engage those who lose interest [60].

Another factor to be considered when designing a health intervention, which was discussed in the workshops, is ensuring that the health-related educational content is tailored to the context and contains learnings that can be applied to the participants’ own lives. Many of the LifeLab activities discussed in the co-design workshops utilised contextually relevant vignettes previously developed using data gathered from a similar demographic [36]. These vignettes are short stories which enable participants to engage in discussions around individuals they would ‘recognise’ or relate to from their community with varying levels of HL. Vignette methodology is used to explore attitudes, values and perceptions of health issues, and other sensitive or controversial topics [61]. They allow the participants to engage in controlled discussion around a relevant and realistic character, whereby individuals can differentiate from themselves, discuss their opinions on a given topic and identify in a non-threatening manner. The use of vignettes in the workshops was very effective, with participants actively engaging in the content and even requesting that more vignettes be utilised in future LifeLab activities. Although the vignettes employed were validated with this population previously [36], written vignettes may still act as a barrier for engagement, given the low literacy levels of the targeted adolescents [40]. It is, therefore, important to consider the methods used to present the vignettes in order to improve accessibility. Viable methods to remove this barrier, which have been demonstrated in previous research, may be the use of audio-visual [62] or digital [63] versions of the peer vignettes. The discussions around the vignette’s lifestyles and health behaviours provided rich insights into the challenges that adolescents from this cohort face and allowed the participants to identify areas of learning that not only interested them, but that they felt would help tackle the HL-related challenges that they and their peers encounter in their everyday lives. Some of the suggestions made by participants for future learning in LifeLab included educational content around the impact of lifestyle behaviours on the body’s appearance and its ability to function; ‘good v bad’ lifestyle behaviours; and the influence of social media on young people. Although ‘less is better’ is often suggested in relation to screentime [64], today’s young people are digital natives, battling with the overabundance of (valid and invalid) information and material available at their fingertips [65] and navigating new health risk behaviours [66] associated with screens. Yet alongside this, technology could be a way to facilitate the preferred learning engagement strategies and overcome some of the practical and logistical considerations cited by participants in this study. There is, therefore, a need to balance these considerations to develop functional, interactive and critical health literacy for participants within LifeLab.

This study highlights the benefit of adopting a co-design approach, whereby key stakeholders have the opportunity to create a health intervention that is meaningful to their context, is equity driven, and is sustainable. It is recognised that too often interventions are based solely on academic theory and lack the contextual understanding required [67]. This has led to a surge in interventions utilising a participatory approach in recent years. The value in such an approach is highlighted throughout the findings of this study, where participants provided invaluable feedback on the learning activities and overall intervention. Moreover, it has been stated that involving socioeconomically disadvantaged adolescents in the design of a health intervention not only increases the likelihood of producing an efficacious intervention that meets the needs of the targeted population [68], but also provides designers with a sense of ownership which can empower them to improve their lifestyle [69]. The WHO are strong advocates for involving peers in the design of health intervention, particularly in ‘hard to reach’ populations, as they believe it contributes to greater acceptability and provides a sense of leadership [13].

## 5. Limitations

Findings of the study aside, it is not without limitations. The COVID-19 pandemic caused major disruptions to the project. First, this study was intended to be carried out in person, rather than remotely, allowing participants to physically trial the learning activities before engaging in co-design workshops. Due to local government restrictions, this was not possible and therefore required us to pivot to remote methods, such as Zoom and Microsoft Teams. This led to challenges in describing the ideas for the learning activities as well as the concept of LifeLab, possibly resulting in some confusion from participants. In order to clarify our intentions, the facilitators of the workshops used clear and concise PowerPoint presentations to describe the learning activity; designed online versions of the practical activities for the participants to engage with; and constantly checked for understanding to ensure that the participants grasped the station/activity content. Despite these efforts, it is very difficult to replicate an in-person experience online. In addition, this was the research teams’ first time gathering data using this online methodology, so, as to be expected, it was not without hiccups. Furthermore, COVID-19 impacted the recruitment of participants. As a result of the subsequent pressures on schools, three of the four previously recruited schools opted out of this phase of the study. Although the YHF were consequently recruited, the total sample was still relatively small.

## 6. Conclusions

The current paper details the co-design of an engaging HL intervention targeting socioeconomically disadvantaged adolescents. The findings highlight key strategies for engaging young people in HL learning, practical and logistical considerations when implementing an engaging HL intervention, and content that adolescents from this populations feel is meaningful and valuable to learn about. The methodology undertaken provides key stakeholders with the opportunity to develop a contextually tailored intervention that has the potential to tackle health inequalities in this population. Participants co-designed multiple interactive learning activities which are aimed at improving HL across various health domains. While the findings are most directly relevant to the LifeLab intervention itself and will directly inform its content and structure, there are learnings from this study which can be applied by teachers and researchers alike in developing educational content. In addition, findings support the importance of the methodological approach taken, an approach which can be replicated across other demographics in order to refine elements over time and also to co-design and formatively evaluate future interventions.

## Figures and Tables

**Table 1:** Themes and Sub-themes.

Theme	Sub-Theme	Example Quote
Preferred Learning Engagement Strategies	Healthy competition	*I think if you even like put the groups against each other… it would make it like a bit competitive and then everyone would want to do it, as most people are competitive (YHF participant)*
	Interactive tasks	*Yeah, it was good; it was fun as well to be interactive and you weren’t waiting around for long (YHF participant)*
	Problem solving	*Will there be like obstacles on it? Is it going to be almost like a maze? (School A participant)*
	Providing variety and choice	*I liked how there was like different sections that you could choose (YHF participant)*
Practical & Logistical Considerations	Amount of time per learning activity	*It would probably be best to make it around 10 min, even longer maybe (School A participant)*
	Number of students per activity	*I don’t know, it depends on how big the group is. 10 people is gonna be kinda hard to get everyone (involved), but if it’s 5/6 it’s easy to get a smaller group to work together on one thing. If it’s bigger, people are being left out (YHF participant)*
	Creating the appropriate physical space	*I thought the colours and the objects in the room were really important (YHF participant)*
Ideas for LifeLab Content	Relating the content and learnings to real life	*I think seeing the effect of a lack of sleep on the vignettes because even though they’re fictional, it’s probably not too far off from some people in the class. So that would maybe, like, make them [think]… oh, maybe I shouldn’t be doing that (YHF participant)*
	The influence of social media on young people	*He (the vignette) is believing stuff on Instagram that could be fake (School A participant)*
	Lifestyle behaviours and their impact on health	*I think it’s important to show, like, how your choices now, even though they might be small, and they might not seem significant, how they can, like, build up in your body (School A participant)*

## Data Availability

Not applicable.

## Supplementary Materials

The following supporting information can be downloaded at: https://www.mdpi.com/article/10.3390/children9081230/s1, Figure S1: Flow diagram for participant recruitment.
