# Supplementary figures and images for: LifeLab: Co-Design of an Interactive Health Literacy Intervention for Socioeconomically Disadvantaged Adolescents’
Source: Children (Basel). 2022 Aug 15;9(8):1230. doi: 10.3390/children9081230 (PMC9406774; doi:10.3390/children9081230)

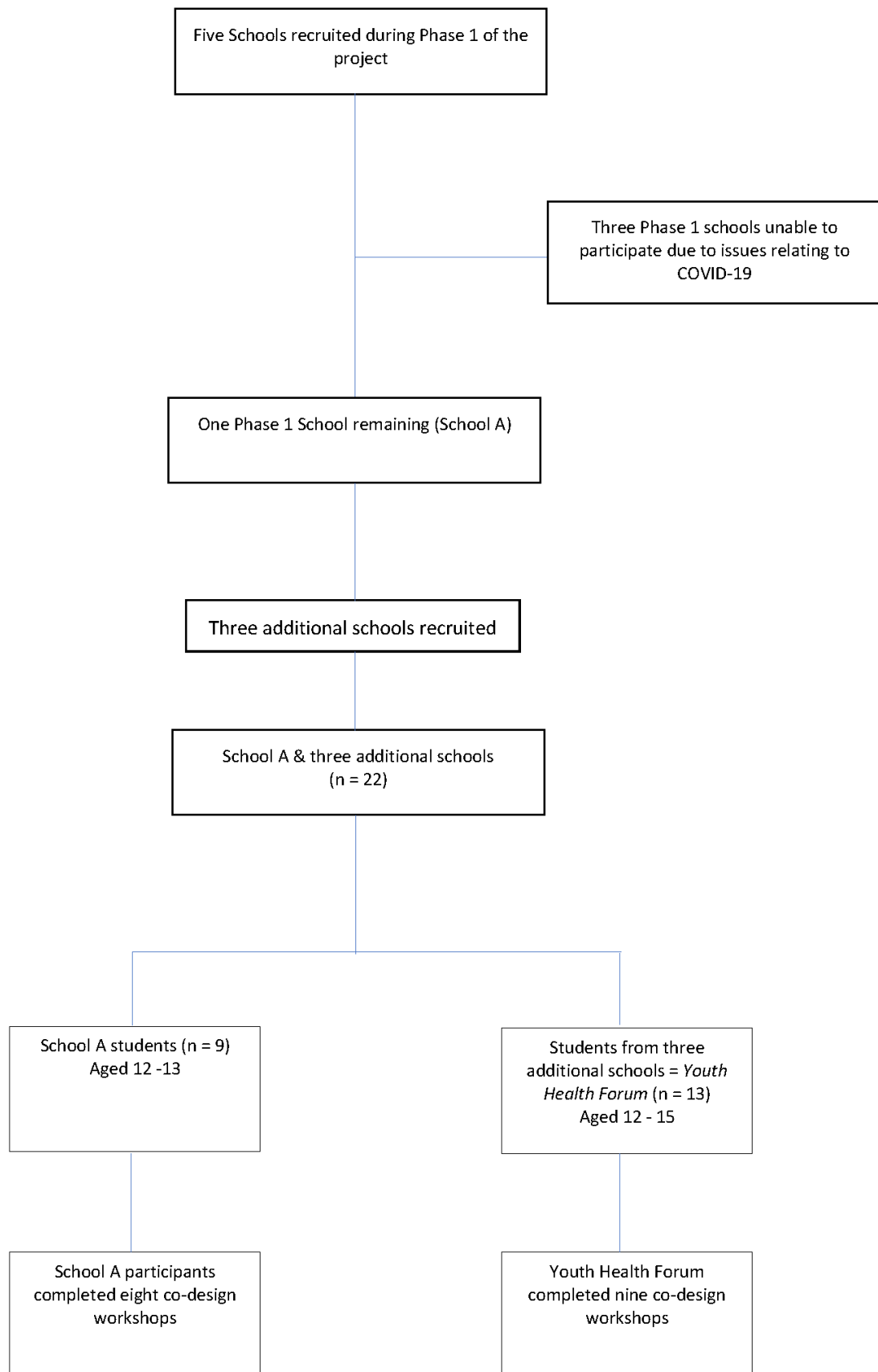

Figure S1: Flow diagram for participant recruitment.

Supplement: Supplementary file 1 [file children-09-01230-s001.zip › children-1822234-supplementary.pdf]
